# Management of unpredictable outcomes of costochondral grafts

**DOI:** 10.1016/j.ijscr.2019.07.074

**Published:** 2019-08-16

**Authors:** Mmathabo G. Sekhoto, Risimati E. Rikhotso, Sumetha Rajendran

**Affiliations:** a Chris Hani Baragwanath Hospital, Division of Maxillofacial and Oral Surgery, University of the Witwatersrand, 26 Chris Hani Road, Diepkloof 319-lq, 1860, Johannesburg, South Africa; b Queen’s Hospital, United Kingdom

**Keywords:** Case report, Maxillofacial, Trauma, Paediatric, Temperomandibular joint

## Abstract

**Introduction:**

Temporomandibular joint (TMJ) ankylosis is a debilitating mandibular hypomobility disorder that may be associated with serious functional and aesthetic deformities including limited mouth opening, impaired mastication and disturbances of facial and mandibular growth. The ultimate treatment goal of TMJ ankylosis is to restore altered joint mechanics, correct associated dentofacial deformities and prevent reankylosis.

**Presentation of case:**

We present a case of a 16 year old patient who returned with excessive mandibular growth and TMJ reankylosis following treatment of TMJ ankylosis with CCGs when he was 8 years old.

**Discussion:**

Various treatment modalities for TMJ ankylosis have been described. These include gap arthroplasty (GAP), interpositional gap arthroplasty (IPG) and/or total joint reconstruction (with autogenous costochondral grafts or alloplastic materials), and distraction osteogenesis. Costochondral grafts (CCGs) are generally preferred for the treatment of TMJ ankylosis, especially in the growing patient. Their advantages include biocompatibility, no financial cost to the patient, low donor site morbidity, and most importantly their potential for mandibular growth. The growth of the mandible is however unpredictable.

**Conclusion:**

The optimal surgical management of this complex patient with combined orthognathic surgery and total alloplastic joint reconstruction is discussed.

## Highlights

• TMJ ankylosis is a debilitating mandibular hypomobility disorder.
• CCGs are generally considered as the gold standard for autogenous reconstruction of mandibular condyle.
• Total alloplastic TMJ prosthesis is a viable option in the case of undesirable outcomes from CCGs.
• Management of TMJ ankylosis in children is dependent on aggressive physiotherapy, and long-term follow-up.

## 1 Introduction

This work has been reported in line with the SCARE criteria [1].

Temporomandibular joint ankylosis is defined as hypomobility of the mandible resulting from fibrous or bony union of the mandibular condyle to the cranial base. Aetiological factors include trauma, infections, pathological processes and degenerative diseases [2]. Trauma is reported to be the most common predisposing factor to TMJ ankylosis [[3], [4], [5]]. Early management of condylar fractures (particularly the intracapsular types) in children is challenging because injury to the joint may not be diagnosed immediately or may be missed completely until years later when there is restriction in mouth opening and TMJ ankylosis. Early aggressive physiotherapy and long-term follow-up are crucial if the undesirable sequelae of this condition are to be avoided.

Sawhney classified TMJ ankylosis into 4 groups: Type 1 – fibrous adhesion all around joint; Type 2 – bony fusion of the condyle head to the articular surface, limited to the outer edge of the joint; Type 3 – bony bridge between the mandibular ramus and the zygomatic arch and Type 4 – the entire joint is replaced by the bony ankylotic mass [6].

TMJ ankylosis in skeletally growing children results in mandibular deformity and growth impairment, masticatory and speech problems, poor oral hygiene causing dental caries and periodontal disease, as well as facial asymmetry which worsen with ongoing growth [7,8]. Furthermore, it has a negative impact on the psychosocial development of the patient due to obvious facial deformity.

Management of TMJ ankylosis and associated facial deformity in children is very difficult and complex. Not only is surgical correction challenging and technically difficult, but the surgeon must always consider the high incidence of recurrence and potential effects of the treatment on growth [9]. Treatment of TMJ ankylosis can be carried out through gap arthroplasty (GAP), interpositional gap arthroplasty (IPG), joint reconstruction (with autogenous costochondral grafts or alloplastic materials) or distraction osteogenesis.

Costochondral grafts (CCGs) have been used for many years in reconstruction of temporomandibular joint and are generally considered as the gold standard for autogenous reconstruction of mandibular condyle. They are favoured because of their biologic and adaptive behaviour, particularly in skeletally immature individuals [3]. However, CCGs have the disadvantage of unpredictable growth pattern. CCGs can grow normally, overgrow or undergrow as well as undergo ankylosis or complete resorption.

## 2 Case report

A 16-year old male patient consulted the maxillo-facial and oral surgery outpatient clinic of the Chris Hani Baragwanath Academic Hospital (Johannesburg, South Africa) complaining of severe facial deformity and inability to open his mouth. The patient’s history was significant for bilateral post-traumatic TMJ ankylosis sustained at the age of 4 years. He was treated with gap arthroplasty and bilateral costochondral grafts at the age of 8 years. Thereafter, his mouth opening gradually decreased and multiple jaw stretching procedures were performed under general anaesthesia.

**Fig. 1:**
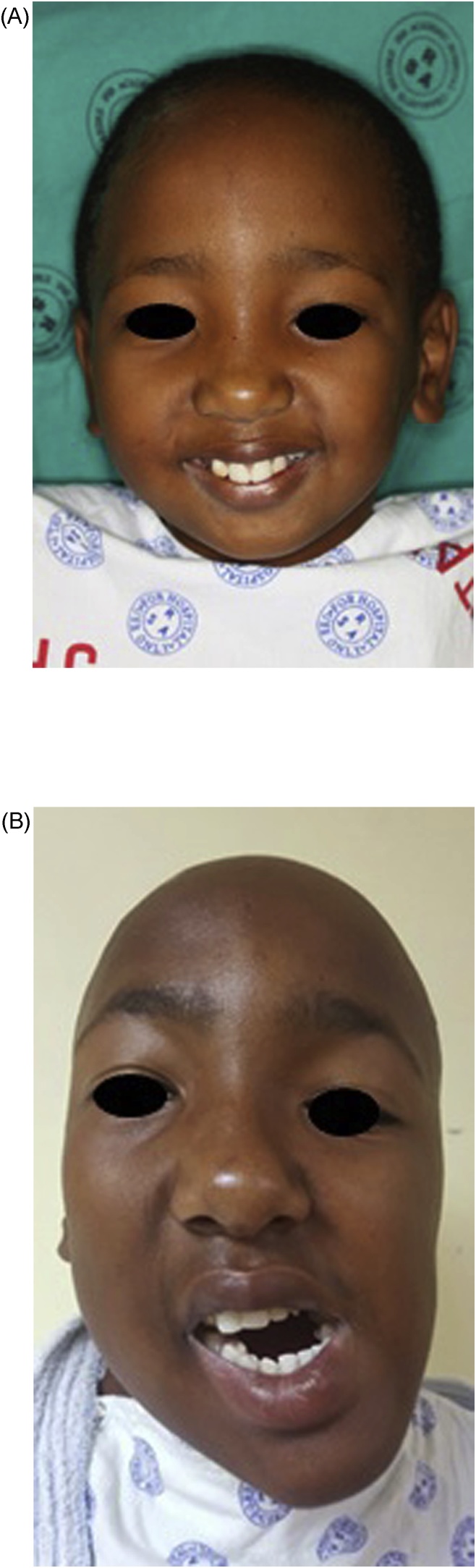
(A) Patient at the age of 8 years. (B) Patient at the age of 16 years.

Five years later, chin asymmetry and concomitant malocclusion became apparent and subsequent diagnosis of right CCG overgrowth and reankylosis on the left side was made. A gap arthroplasty was performed on the left side, however the patient returned a year later with bilateral TMJ reankylosis (Fig. 1).

**Fig. 2:**
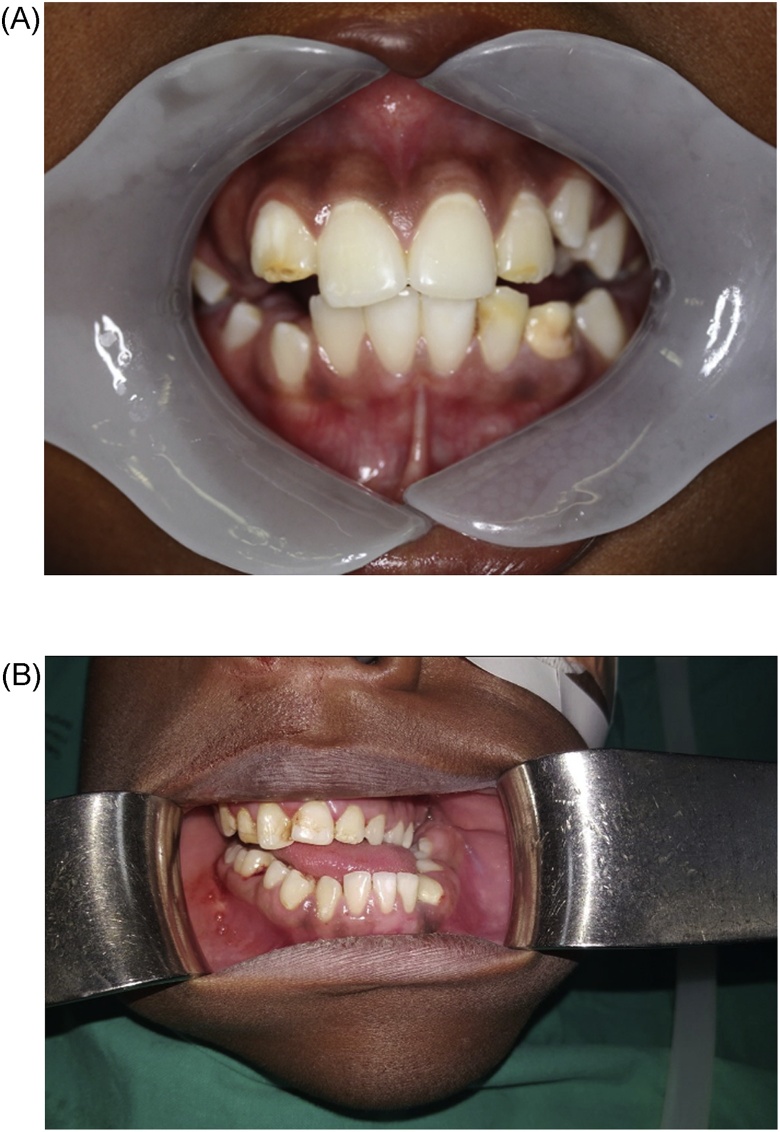
(A) Pre-operative attempted mouth opening at age 8 years. (B) Pre-operative attempted mouth opening at age 16 years.

The clinical examination revealed facial asymmetry with deviation of the mandibular midline to the left. His mouth opening was restricted to approximately 5 mm interdental distance (IDD). He also presented with a cant in the occlusal plane and marked malocclusion. Fig. 2A and B show pre-operative mouth opening at 8 year and 16 years.

**Fig. 3:**
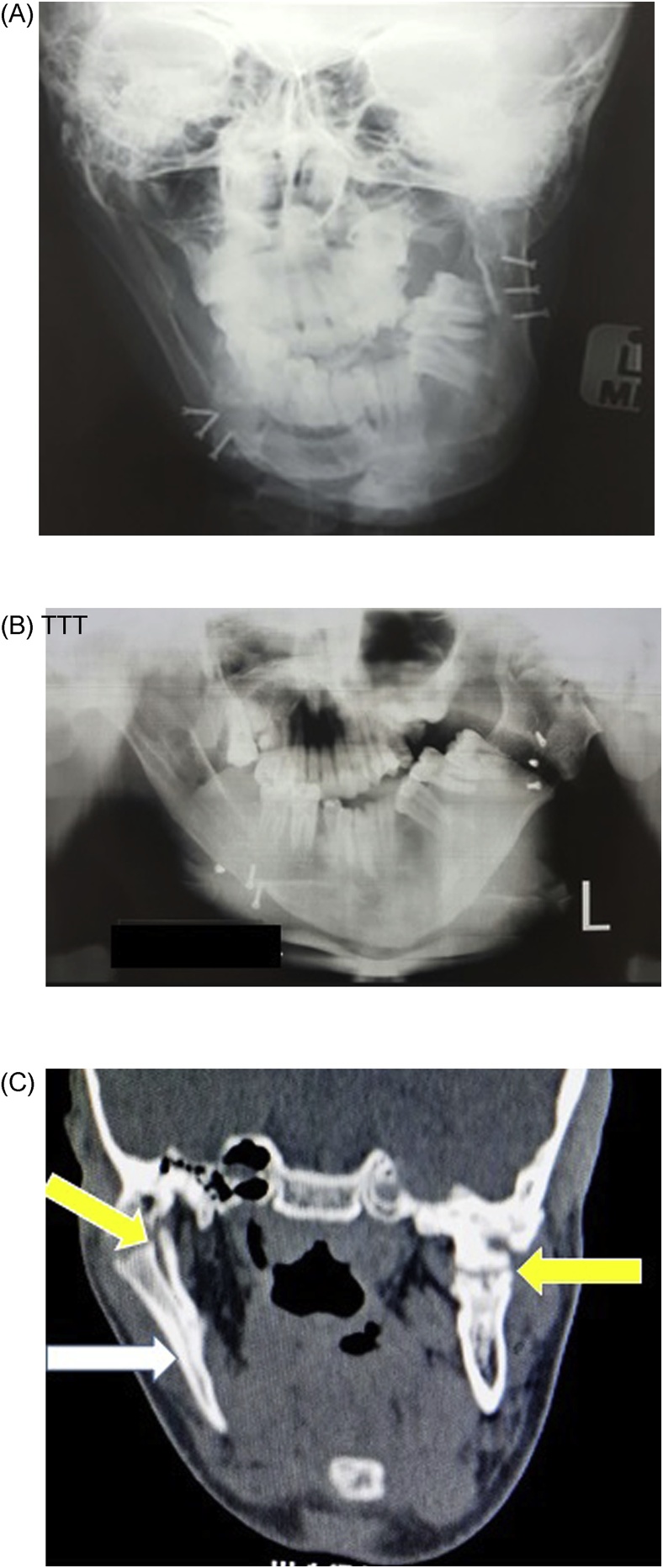
(A) Pre-operative radiographs at age 16 years: postero-anterior (mandible). (B) Orthopantomography. (C) Coronal CT scan.

Radiographic investigation included PA mandible (Fig. 3A), orthopantomogram (Fig. 3B) and coronal CT Scans (Fig. 3C). These images confirmed elongation of the vertical ramus on the right side, asymmetry of the chin towards the left side and bilateral ankylosis of the TMJ (yellow arrows) with right mandibular overgrowth (white arrows) respectively.

**Fig. 4:**
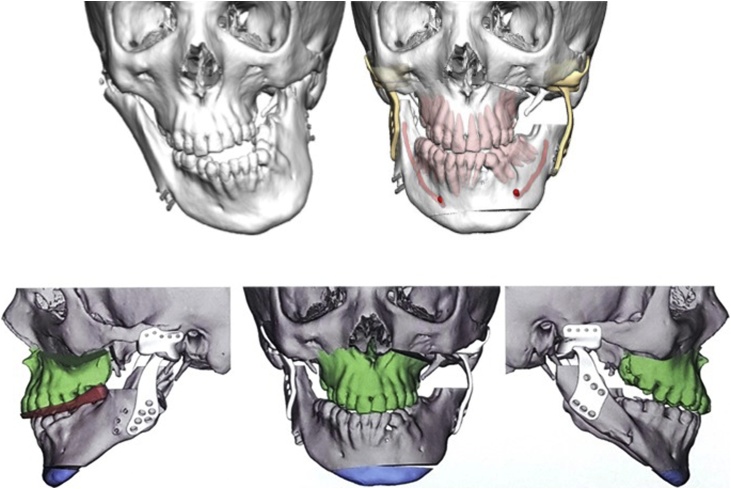
3D images showing preoperative image and Virtual Surgical Planning for orthognathic surgery and custom joints.

**Fig. 5:**
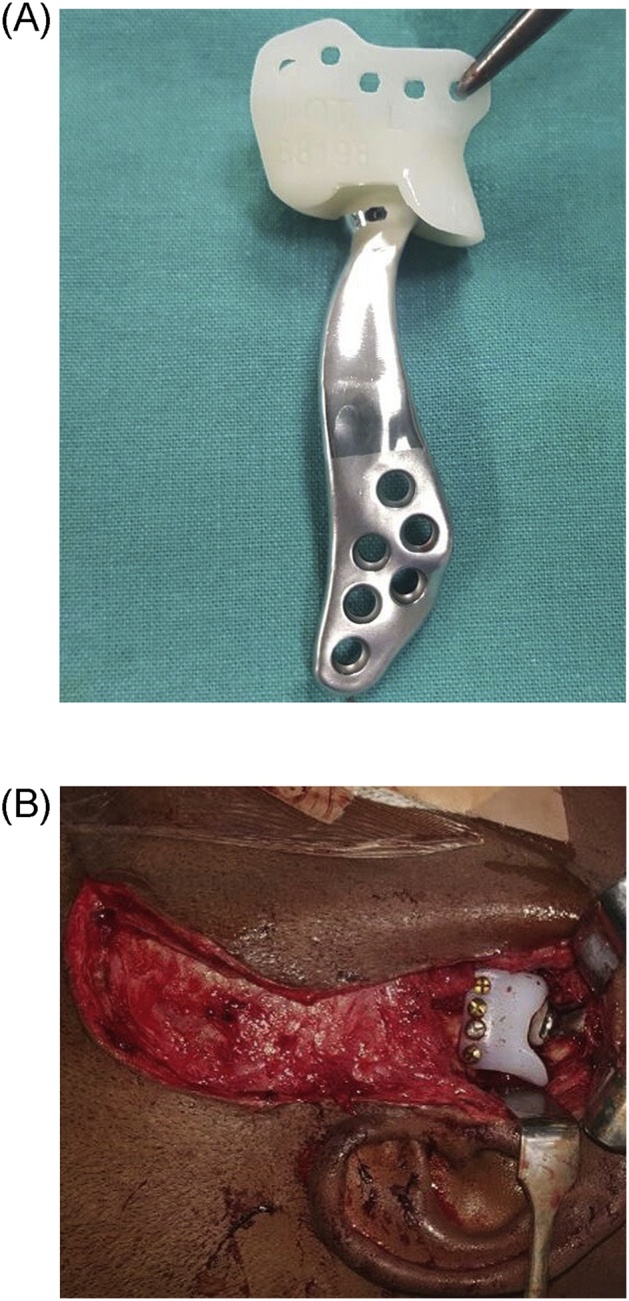
(A) Biomet Microfixation patient-matched alloplastic TMJ prostheses. (B) Intra-operative image of placement of TMJ prostheses.

**Fig. 6:**
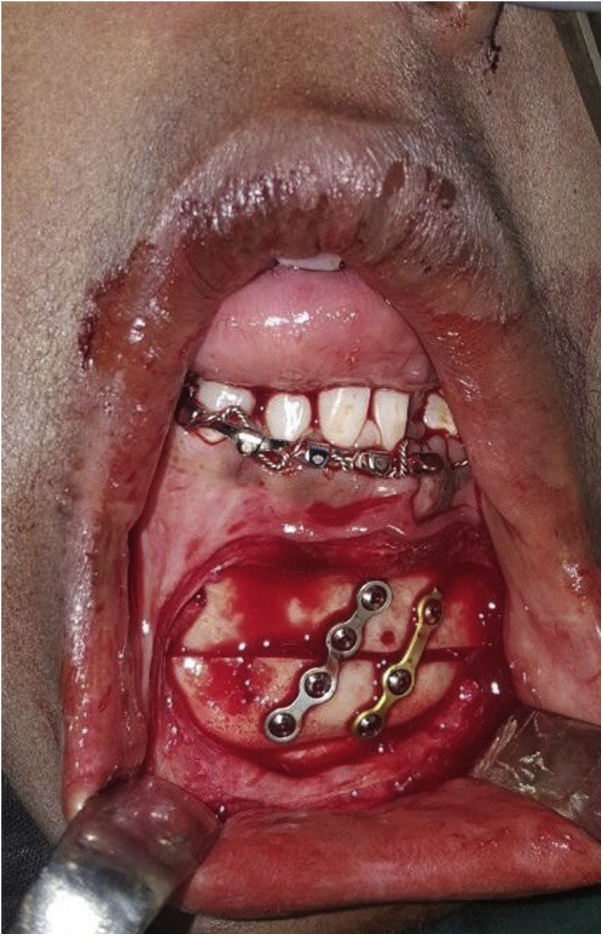
Intra-operative image of genioplasty.

In view of the severity of the deformity, combined orthognathics and TMJ reconstruction with custom joints was planned. The procedure and custom-made prostheses were planned using 3D imaging and stereolithic model generated from his CT scan (Fig. 4). Surgical release of bilateral ankylotic masses was carried out using surgical guides via Al-Kayat and Bramley (modified preauricular) and Risdon submandibular approach. Temporomandibular joints were reconstructed with Biomet Microfixation patient-matched alloplastic TMJ prostheses (Fig. 5). An IDD of 35 mm was achieved. Facial asymmetry, occlusal plane cant and cross-bite were corrected with Le fort I osteotomy and a genioplasty (Fig. 6).

**Fig. 7:**
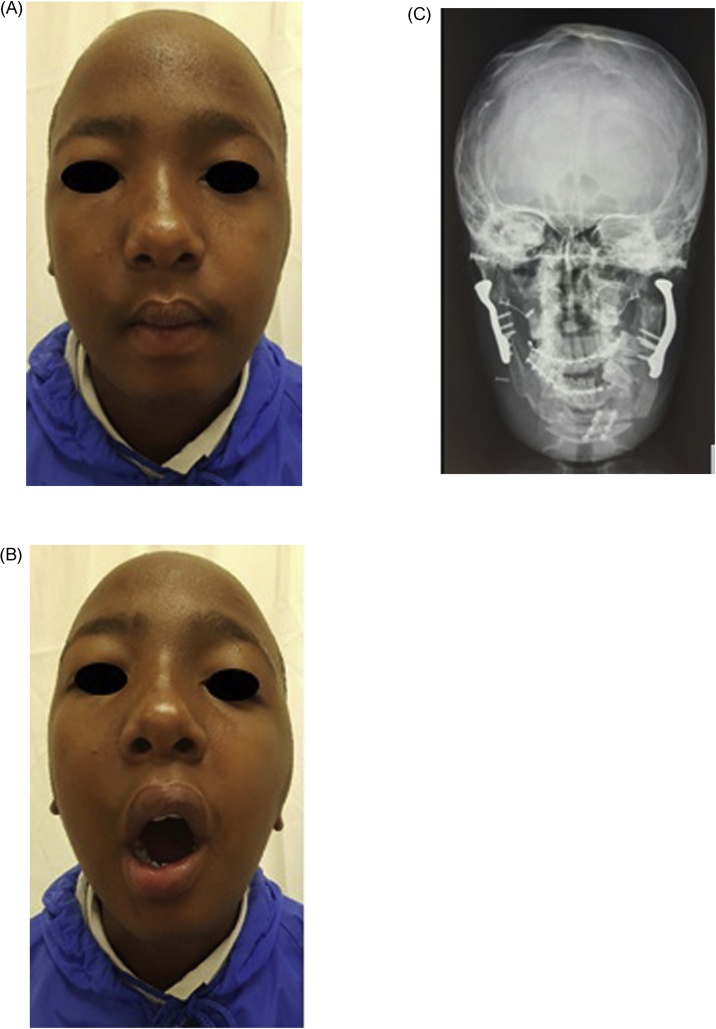
(A) Post-operative images depicting good facial symmetry. (B) Good maximum mouth opening. (C) Radiograph showing alloplastic joint prostheses.

At the time of his 12 month postoperative evaluation, he had a maximum mouth opening of 30 mm IDD and his functional and aesthetic evaluation was satisfactory (Fig. 7).

## 3 Discussion

According to Ware et al. the mandibular condyle acts as a growth centre and plays a crucial role in maintaining the facial skeletal growth [10]. Their study demonstrated that the growth of the mandible almost resembled that of the normal mandible following replantation of the excised condyle, when compared to cases where the condyle was either removed or displaced. To maintain normal mandibular form and function, TMJ reconstruction should be undertaken as soon as the diagnosis of condylar or TMJ pathology is made.

An ideal total TMJ reconstruction, whether autogenous or alloplastic, is one that closely resembles and replaces the form and function of the original joint. Regardless of which material is used, the ultimate management goals remain to (1) improve TMJ function and facial form, (2) reduce pain and disability, (3) prevent excessive treatment and cost, and (4) avoid further morbidity [11].

Autogenous grafts used in reconstruction of TMJ have evolved over the years. Several grafts including metatarsophalangeal joint, costochondral graft, fibular head, iliac bone graft, clavicle and sternoclavicular joint have all been used in an attempt to find an ideal graft. The costochondral graft (CCG), since its description by Gillies in 1920 [12], has over time gained universal endorsement for replacement of the ramal condylar unit in ankylosis, and in patients with end-stage joint disease in general.

CCG has gained popularity because of its three important functional parts: the bony part which is used to manage the height of the ramus of the mandible and fixation of the graft, the cartilaginous part which acts as an interpositional material, and the growth centre which lies between the bony and the cartilaginous part [13,14].

This technique has for years been revised and subjected to scientific scrutiny. The purported advantages of CCG include ease of harvest and adaptation, minimum morbidity, numerous donor sites, regeneration of the donor site and retention of integrity after transplantation. Also, in the growing patient, CCG with intact costochondral junction not only provides an excellent choice of autogenous tissue for replacement of a missing, malformed and or diseased condyle but also demonstrates growth potential [9,15].

Kaban et al. and Ware and Brown have shown that CCG have proved the best among the autogenous tissue for reconstruction of the mandibular ramal condylar unit [15,16]. The present case was initially correctly managed with CCGs at the age of 8 years to restore both the form and function of TMJ and to provide him with a graft that will keep up with his facial growth demand. However, a systemic review by Kumar et al. (2015) confirmed the unpredictability of CCGs outcomes, particularly when used in children [17]. Some CCGs show favourable growth, while others could undergo complete resorption, undergrowth, overgrowth or reankylosis. They concluded that the growth potential associated with CCGs lacked scientific evidence.

This case demonstrated right costochondral graft overgrowth and subsequently bilateral TMJ reankylosis which were noticed 4 years after CCGs. Subsequently, significant facial asymmetry developed due to both increased vertical ramus height on the right-side and a retarded growth and reankylosis on the left-side. Reankylosis was probably related to lack of long-term compliance with physiotherapy. The disturbance of vertical ramus growth resulted in obvious asymmetry of facial skeleton. This observation is particularly true in growing patients [10]. Facial deformities vary in severity and depend on the age of onset and age at the time of reconstruction. The deformity in the present case also coincided with his growth spurt, which presumably was responsible for its severity.

Total alloplastic TMJ prosthesis was deemed the treatment modality of choice for this patient because autogenous grafts, namely CCGs had already yielded undesirable outcomes. TMJ prosthesis was chosen because of the lack of a secondary donor site, a shorter surgical time and the return to immediate function. The use of patient-fitted total TMJ alloplastic prosthesis offers additional better outcomes particularly in patients with severe dentofacial deformities such as ours because, not only will it replace and reconstruct the joint and the deficient ramus component, but it also assists in correcting aesthetic discrepancies [18].

Orthognathic surgery and the custom joints successfully rehabilitated our patient, combined with intense physiotherapy. The severe hypomobility in this case precluded presurgical orthodontics. Orthodontic treatment is still required to correct the severe malocclusion resulting from dental compensation for the skeletal discrepancies.

## 4 Conclusion

Management of TMJ ankylosis in children is very challenging and technically demanding. It relies entirely on continued patient-dependant aggressive physiotherapy, long-term follow-up and a stepwise interceptive planning and approach.

This case illustrates that although CCGs are considered the gold standard for autogenous reconstruction of mandibular condyle, their unpredictable growth pattern often yield suboptimal outcome, resulting in severe facial deformities. TMJ prosthesis proved to be a viable non-autogenous option for correction of the severe malocclusion and skeletal discrepancy.

To date, complications such as prosthesis fracture or screw loosening have not been observed in this patient after a 2-year follow-up. Long-term periodic follow-up in the form of clinical examination and radiographic evaluation is mandatory to validate these promising results.

## Funding

None.

## Ethical approval

Exempt from ethical approval.

## Consent

Written informed consent was obtained from the parent for publication of this case report and accompanying images. A copy of the written consent is available for review by the Editor-in-Chief of this journal on request.

## Author’s contribution

Dr. Mmathabo Sekhoto – data curation, formal analysis, project roles/writing – original draft.

Dr. Risimati Rikhotso – conceptualisation, data curation, methodology.

Dr. Sumetha Rajendran – data curation, project role/ writing – review and editing.

## Registration of research studies

Not applicable.

## Guarantor

Dr Risimati Rikhotso.

## Provenance and peer review

Not commissioned, externally peer-reviewed.

## Declaration of Competing Interest

No conflict of interest.
